# The effect of *Mycobacterium tuberculosis* treatment on thrombelastography-assessed haemostasis: a prospective cohort study

**DOI:** 10.1186/s12959-024-00625-4

**Published:** 2024-06-26

**Authors:** Hans Johan Niklas Lorentsson, Christina R. Clausen, Daniel Faurholt-Jepsen, Katrine Bagge Hansen, Sidse Graff Jensen, Rikke Krogh-Madsen, Per G. Hagelqvist, Pär I. Johansson, Tina Vilsbøll, Filip K. Knop, Pernille Ravn

**Affiliations:** 1 Section of Infectious Diseases, Department of Medicine, Herlev and Gentofte Hospital, University of Copenhagen, Hellerup, Denmark; 2 Center for Clinical Metabolic Research, Herlev and Gentofte Hospital, University of Copenhagen, Hellerup, Denmark; 3 Clinical Research, Steno Diabetes Center Copenhagen, Herlev, Denmark; 4 Department of Infectious Diseases, Rigshospitalet, University of Copenhagen, Copenhagen, Denmark; 5 Department of Clinical Medicine, Faculty of Health and Medical Sciences, University of Copenhagen, Copenhagen, Denmark; 6 Section of Respiratory Diseases, Department of Medicine, Herlev and Gentofte Hospital, University of Copenhagen, Hellerup, Denmark; 7 Department of Infectious Diseases, Hvidovre Hospital, University of Copenhagen, Hvidovre, Denmark; 8 Center for Physical Activity Research, Rigshospitalet, University of Copenhagen, Copenhagen, Denmark; 9 CAG Center for Endotheliomics, Rigshospitalet, University of Copenhagen, Copenhagen, Denmark; 10 Section of Infectious Diseases, Department of Medicine, Herlev and Gentofte Hospital, Hellerup Hospitalsvej 1, +45 38 67 38 67, Hellerup, 2900 Denmark

**Keywords:** Cardiovascular disease, Inflammation, Thrombelastography, Tuberculosis infection, Tuberculosis disease

## Abstract

**Background and objective:**

Tuberculosis disease (TB) and tuberculosis infection (TBI) have been associated with increased risk of cardiovascular disease which may be connected to infection-related haemostatic changes. It is unknown if treatment of *Mycobacterium tuberculosis* influences haemostasis. Here, we assessed if TB or TBI treatment affects thrombelastography (TEG)-assessed haemostasis.

**Methods:**

Individuals with TB or TBI were included from a TB outpatient clinic in Copenhagen, Denmark. Patients treated with antithrombotic medication or systemic immunosuppressants were excluded. TEG analysis was performed before and after TB/TBI treatment using the TEG^®^6s analyser to provide data on the reaction time of clot initiation (R) (min), the speed of clot formation (K) (min) and clot build-up (Angle) (°), maximum clot strength (MA) (mm), and clot breakdown/fibrinolysis (LY30) (%). Differences in TEG were assessed using paired *t* tests.

**Results:**

We included eleven individuals with TB with median [interquartile range] [IQR] age 52 [32] years and mean (standard deviation) (SD) body mass index (BMI) 24.7 (6.3) kg/m^2^ as well as 15 individuals with TBI with median [IQR] age 49 [25] years and BMI 26.0 (3.2) kg/m^2^. Treatment reduced MA for both TB (64.0 (6.3) vs. 57.9 (5.2) mm, *p* = 0.016) and TBI (61.3 (4.1) vs. 58.6 (5.0) mm, *p* = 0.023) whereas R, K, Angle and LY30 were unaffected.

**Conclusion:**

TEG analysis showed that treatments of TB and TBI were associated with reduced MA which may indicate the existence of cardiovascular benefits from therapy.

**Trial registration:**

Registered at ClinicalTrials.gov 05 April 2021 with registration number NCT04830462.

**Supplementary Information:**

The online version contains supplementary material available at 10.1186/s12959-024-00625-4.

## Background

Tuberculosis disease (TB), caused by *Mycobacterium tuberculosis* (*Mtb*), is among the deadliest infectious diseases in the world. In 2021, 1.6 million people died from TB, and it is estimated that a quarter of the world’s population would test positive for tuberculosis infection (TBI), which refers to a state of persistent immune reaction to *Mtb* antigens without symptoms of TB [1].

TB and TBI have been associated with an increased risk of cardiovascular diseases (CVD) such as acute myocardial infarction, non-haemorrhagic stroke, and venous embolisms [2–8]. In line with this, data from meta-analyses comparing infected and non-infected individuals have shown that people with TB are 1,5 times more likely to die or suffer from CVD and that TBI more than doubles the risk for coronary artery disease [9, 10]. The effect of TB and TBI treatment on CVD risk is however still unknown.

Shared risk factors for TB and CVD such as smoking and alcohol misuse could drive this association but it is unlikely the sole explanation [11, 12]. TB is an infectious disease characterised by a long incubation time and ongoing inflammation, and despite being asymptomatic by definition, TBI has also been associated with inflammation [13–15]. Inflammation is a known driver of hypercoagulability and CVD as observed in people with chronic inflammation from rheumatological diseases, malignancies or chronic infections such as human immunodeficiency virus (HIV) and hepatitis [16–19]. Furthermore, cytokines such as interferon-gamma (IFN-γ), tumour necrosis factor-alpha (TNF-α) and interleukin-6 (IL-6), are not only associated with TB but also with CVD [20–23]. Thus, the increased CVD risk in persons with TB and TBI may be linked to a state of infection-related inflammation leading to hypercoagulability. This prothrombotic state may limit bacterial spread via vessel occlusion but also acts as a double-edged sword since reduced vascularisation isolates the area from the immune system and enables the accumulation of *Mtb* antigens and host lipids necessary for disease development [24, 25]. As treatment for TB reduces inflammation, we hypothesise that treatment also reduces hypercoagulability in people with TB and TBI [26, 27].

Thrombelastography (TEG) is widely used to monitor and direct haemostatic treatment and captures the different aspects of haemostasis from clot initiation to clot lysis [28]. In recent years, it has been demonstrated that TEG can detect prothrombotic haemostatic alterations in populations with high CVD risk compared to controls and may be a useful tool for predicting CVD risk [28–37].

The aim of this study was to assess if treatment for TB and TBI reduces inflammatory parameters and modulates TEG-assessed haemostasis.

## Methods

### Study design and participants

This study contains data from a purely observational cohort study investigating the immune and metabolic impact of TB treatment and a similar study on TBI treatment (https://clinicaltrials.gov/study/NCT04830462). Participants were consecutively enrolled from the TB outpatient clinic at Herlev and Gentofte University Hospital, Copenhagen, Denmark, between April 2021 and September 2022. Blood sampling and collection of clinical information were performed before (baseline) and after (follow-up) treatment of TB or TBI. In cases where TB treatment was prolonged, follow-up samples were taken after 6 months of treatment.

The inclusion criteria were planned treatment for TB or TBI, age ≥ 18 years and informed consent. The exclusion criteria were known immunosuppression (e.g., HIV infection, oral corticosteroid treatment within 14 days before inclusion, ongoing immune modulating treatment or splenectomy), known contraindication (e.g. allergy) to rifampicin (RIF) and isoniazid (H), active liver disease, severe inflammatory or rheumatological diseases with immune activation and need for prolonged systemic treatment, active malignancy or ongoing chemotherapy, pregnancy or treatment with oral contraception, antibiotic treatment within 2 days (TBI only) or severe infection within 14 days before enrolment (TBI only). An additional exclusion criterion for participation in TEG analysis was the use of antithrombotic medication.

### Diagnosis, treatment and definition of TB and TBI

The diagnosis and treatment of TB and TBI were performed by the attending physician in the TB outpatient clinic according to local and global guidelines [38, 39]. The standard TB treatment consisted of daily H 300 mg + pyridoxin 20 mg, RIF 600 mg, ethambutol 1200 mg and pyrazinamide 2000 mg for two months, followed by H 300 mg and RIF 600 mg for four months. The standard TBI treatment regimens were daily RIF 600 mg for four months or daily H 300 mg for six months. Adjustment for weight, kidney function, and TB severity was done according to Danish guidelines [39].

TB cases were defined by one or more of the following criteria: positive microscopy results with acid-fast bacteria, positive *Mtb* DNA polymerase chain reaction, positive *Mtb* culture or clinical and radiological findings suggestive of TB with clinical and radiological response to TB treatment. TBI cases were defined by a positive interferon-gamma release assay (IGRA) with no clinical or radiological findings suggestive of TB.

### Procedures for collecting general information

Clinical information (e.g., medications, smoking status, comorbidities and alcohol use) was registered at baseline via interviews with the participant or by review of the electronic patient record. Changes in medicine were registered at follow-up.

### Procedures for collecting and analysing biochemical measures

Samples intended for general biochemistry (alanine amino transferase (ALAT), c-reactive protein (CRP), platelet count and international normalised ratio (INR) estimation) were analysed at the local Department of Clinical Biochemistry. Cytokines (IFN-γ, TNF-α, interleukin-1beta (IL-1β) and IL-6) were analysed according to the manufacturer’s instructions. Correlated cytokine samples were analysed on the same plate (V-PLEX Human Proinflammatory Panel I (4-Plex), catalogue number K15052D-2) using the MESO QuickPlex SQ 120 with subsequent analysis in Workbench version 4.0 (Meso Scale Diagnostics, Rockville, MA, USA). Values below the detection limit or with a coefficient variability above 20% were excluded. Only 3 pairs of baseline and follow-up measurements of IL-1β were above the detection limit, therefore, IL-1β was not included in the final analysis. Lower and upper detection limits are presented in supplementary Table S1. For two samples, TNF-α was analysed only as a single agent due to malfunctioning washing equipment. Samples for TEG analysis were drawn in a citrated tube (3.2% trisodium citrate) which was inverted 5 times immediately after sampling and again 5 times just before analysis. After 15 min but within 2 h, the samples were transferred to a standard multichannel cartridge for citrated whole blood and analysed according to the manufacturer’s instructions using the TEG^®^6s (Haemonetics, Boston, MA, USA) [40]. The TEG results used in this study were; citrated kaolin (CK) reaction time (CK R) - time (min) to develop a 2 mm clot amplitude, measures clot initiation speed; CK kinetics (CK K) - time (min) from 2 mm amplitude to 20 mm amplitude, measures early clot kinetics; CK angle – degree (°) of clot strength increase, measures clot kinetics; CK maximum amplitude (CK MA) – maximum amplitude (mm), measures maximum clot strength; CK lysis 30 (CK LY30) – percentage of clot breakdown after 30 min, measures fibrinolysis; and citrated functional fibrinogen maximum amplitude (CFF MA) – maximum amplitude (mm) with inactivated platelets, measures the impact of fibrinogen [41, 42].

### Statistical analysis

The primary outcomes in this study were differences in TEG results and inflammatory markers between baseline and follow-up. Data were entered into Research Electronic Data Capture (REDCap) version 13.1.35 [43]. Normally distributed data are presented as means with standard deviations and non-normally distributed data are presented as medians with interquartile ranges. The differences between baseline and follow-up were analysed using paired *t* tests. Non-normally distributed data were analysed after logarithmic transformation. For non-normally distributed data containing 0, logarithmic transformation was performed by adding 1 to all values. Only participants with a TEG before and after treatment were included in the final analysis. One participant with TB received tranexamic acid at baseline and was therefore not included in the analysis of CK LY30. The *p* values were adjusted for multiple comparisons using the Benjamini-Hochberg false discovery rate method. Two-sided *p* values below 0.05 were considered significant. The statistical analysis was performed in R version 4.1.0.

## Results

### Participants

Of the 51 included participants (24 with TB and 27 with TBI), 11/24 (46%) and 15/27 (56%) had TEG taken at baseline and follow-up and were included in the final analysis (supplementary Figure S1).

Of the participants with TB, 9/11 (82%) had baseline samples taken before treatment start and 10/11 (91%) had follow-up samples taken within 30 days after treatment was stopped or at 6 months if treatment was prolonged. Of the participants with TBI, 14/15 (93%) had baseline samples taken before treatment start and 14/15 (93%) had baseline samples taken within 30 days after treatment was stopped (supplementary Table S2).

Three participants with TB reported a change in medicine between baseline and follow-up: one started oral semaglutide 7 mg × 1, one started subcutaneous teriparatid 20 µg, and one started methylphenidate 30 mg × 2. One participant with TBI started linaclotide 290 mg × 1 during the study.

### Diagnosis and treatment

Clinical characteristics of the study participants can be found in Table 1. Of the participants with TB, 7/11 (64%) were culture positive. All the culture negative TB participants had significant radiological and clinical improvement after treatment. No participants with TBI reported previous TB treatment except one participant who was rescreened due to a new TB exposure and was therefore deemed eligible for participation. All participants reported acceptable drug adherence (minimum 90%). Of the participants with TB, 10/11 (91%) received standard treatment and 1/11 (9%) received a modified version of standard treatment due to adverse reactions (moxifloxacin 400 mg × 1 instead of H). Of the participants with TB, 9/11 (82%) received 6 months of treatment and 2/11 (18%) received treatment for a total of 9 months. Of the participants with TBI, 12/15 (80%) received RIF and 3/15 (20%) received H.

**Table 1:** Clinical characteristics of the participants

	Tuberculosis disease (*n* = 11)	Tuberculosis infection (*n* = 15)
**Characteristics**		
Male sex, n (%)	8 (73%)	6 (40%)
Age (years)	52 [32]	49 [25]
Diabetes	2 (18%)	1 (7%)
Alcohol consumption (units/week)	1.6 (4.2)	3.6 (10.1)
Active smokers n (%)	1 (9%)	0 (0%)
Former smokers n (%)	2 (18%)	0 (0%)
Charlson comorbidity index (points)	0.7 (0.8)	0.8 (1.0)
**Tuberculosis disease**		
**Location**		
Pulmonary tuberculosis disease n (%)	8 (73%)	
Extrapulmonary tuberculosis disease n (%)	3 (21%)	
**Diagnosis**		
Culture positive n (%)*	7 (64%)	
Microscopy, PCR and culture negative but with clinical and radiological improvement after treatment n (%)**	4 (36%)	

Baseline characteristics of participants with tuberculosis disease or tuberculosis infection. Data are presented as means (standard deviation), median [interquartile range] or n (%)

*Drug susceptibility testing was negative in all cases

**One with pulmonary tuberculosis disease and three with extrapulmonary tuberculosis disease (two lymphatic and one pleural)

PCR: polymerase chain reaction

### Thrombelastography and biomarkers

Treatment was associated with significant reductions in CK MA of 6.1 mm (9.5%; from 64.0 to 57.9 mm) and 2.7 mm (4.4%; from 61.3 to 58.6 mm) for TB and TBI, respectively (Tables 2 and 3). IFN-γ was reduced after treatment in both groups. Platelet count, ALAT and BMI were unchanged by treatment.

**Table 2:** Changes in thrombelastography, inflammation markers and other biomarkers after treatment for tuberculosis disease

	Baseline (*n* = 11)	Follow-up (*n* = 11)	*p* value/adjusted *p* value
**Thrombelastography**			
CK R (min)	6.8 (1.5)	6.6 (1.0)	0.700/0.807
CK K (min)	1.3 (0.5)	1.5 (0.4)	0.258/0.408
CK Angle (°)	73.5 (5.2)	71.1 (3.7)	0.122/0.245
CK MA (mm)	64.0 (6.3)	57.9 (5.2)	0.001/**0.016**
CK LY30 (%) †*	0.1 [0.8]	0.8 [1.0]	0.074/0.202
CFF MA (mm)	29.1 (15.2)	21.3 (6.8)	0.025/0.093
**Inflammation**			
CRP (mg/L) †	9.0 [28.5]	4.0 [0.5]	0.021/0.123
Procalcitonin (µg/L)	0.1 (0.1)	0.0 (0.0)	0.244/0.407
IFN-γ (pg/mL) †	38.3 [53.2]	6.4 [6.8]	< 0.001/**0.009**
TNF-α (pg/mL) †	1.3 [0.4]	0.9 [0.4]	0.023/0.113
IL-6 (pg/mL)	2.5 (2.0)	1.1 (0.9)	0.036/0.121
**Other**			
Platelet count (10^9^/L)	337.2 (174.3)	248.6 (53.8)	0.098/0.227
ALAT (U/L)	34.8 (39.9)	25.8 (12.9)	0.342/0.447
INR	1.0 (0.1)	1.0 (0.1)	0.104/0.222
BMI (kg/m²)	24.7 (6.3)	25.5 (6.1)	0.270/0.402

Normally distributed data are presented as means (standard deviation) and analysed with paired *t* tests. Non-normally distributed data (†) are presented as medians [interquartile range] and analysed using paired *t* tests after logarithmic transformation. The *p* values were adjusted for multiple comparisons using the Benjamini-Hochberg false discovery rate method and are shown as non-adjusted/adjusted with significant adjusted *p* values in bold. Due to missing data, paired *t* tests were performed on less than 11 pairs for: Procalcitonin (*n* = 7) and INR (*n* = 9). *One participant with tuberculosis received tranexamic acid at baseline and was therefore not included in the analysis of LY30. The *p* value represents the difference between baseline and follow-up

CK: citrated kaolin; R: reaction time; K: kinetics; MA: maximum amplitude; LY30: lysis after 30 min; CFF MA: citrated functional fibrinogen maximum amplitude; CRP: C reactive protein; IFN-γ: interferon-γ; TNF-α: tumour necrosis factor-α; IL-6: interleukin 6; ALAT: alanine amino transferase; INR: international normalised ratio; BMI: body mass index

**Table 3:** Changes in thrombelastography, inflammation markers and other biomarkers after treatment for tuberculosis infection

	Baseline (*n* = 15)	Follow-up (*n* = 15)	*p* value/ adjusted *p* value
**Thrombelastography**			
CK R (min)	6.0 (1.5)	6.0 (1.4)	0.969/0.999
CK K (min)	1.4 (0.4)	1.4 (0.4)	0.792/0.848
CK Angle (°)	72.6 (3.4)	72.6 (3.3)	0.974/0.974
CK MA (mm)	61.3 (4.1)	58.6 (5.0)	0.002/**0.023**
CK LY30 (%) †	0.2 [0.6]	0.3 [1.8]	0.062/0.188
CFF MA (mm)	21.2 (4.2)	20.5 (1.3)	0.403/0.483
**Inflammation**			
CRP (mg/L) †	4.0 [0.0]	4.0 [0.0]	0.172/0.304
Procalcitonin (µg/L)	0.0 (0.0)	0.0 (0.0)	0.336/0.458
IFN-γ (pg/mL) †	9.9 [10.5]	4.6 [2.0]	0.003/**0.024**
TNF-α (pg/mL) †	0.8 [0.2]	0.8 [0.3]	0.392/0.490
IL-6 (pg/mL)	0.8 (0.4)	0.6 (0.4)	0.024/0.102
**Other**			
Platelet count (10^9^/L)	258.9 (47.9)	236.6 (43.6)	0.077/0.192
ALAT (U/L)	23.8 (9.6)	25.0 (14.4)	0.740/0.818
INR	1.1 (0.1)	1.0 (0.1)	0.137/0.257
BMI (kg/m²)	26.0 (3.2)	25.4 (3.8)	0.324/0.463

Normally distributed data are presented as means (standard deviation) and analysed with paired *t* tests. Non-normally distributed data (†) are presented as medians [interquartile range] and analysed using paired *t* tests after logarithmic transformation. The *p* values were adjusted for multiple comparisons using the Benjamini-Hochberg false discovery rate method and are shown as non-adjusted/adjusted with significant adjusted *p* values in bold. Due to missing data, paired *t* tests were performed on less than 15 pairs for: CRP (*n* = 14), Procalcitonin (*n* = 14), Platelet count (*n* = 14), ALAT (14), INR (*n* = 12) and BMI (*n* = 13). The *p* value represents the difference between baseline and follow-up

CK: citrated kaolin; R: reaction time; K: kinetics; MA: maximum amplitude; LY30: lysis after 30 min; CFF MA: citrated functional fibrinogen maximum amplitude; CRP: c-reactive protein; IFN-γ: interferon-γ; TNF-α: tumour necrosis factor-α; IL-6: interleukin-6; ALAT: alanine amino transferase; INR: international normalised ratio; BMI: body mass index

## Discussion

We explored the effects of treatment on haemostasis in people with TB and TBI and found that treatment was associated with reduced maximum clot strength and IFN-γ in both groups.

The reduction in CK MA presented here is in agreement with the findings of previous work in which other modalities were used to measure platelet activity in relation to TB [44–46]. These studies have demonstrated that platelet factor-4, which is released from platelets during aggregation, is upregulated in people with TB and that it is normalised by TB treatment [44, 46]. Similarly, Turken et al. reported that platelet reactivity, plasma fibrinogen, factor VIII, antithrombin III and protein C reverted to normal levels after 4 weeks of TB treatment [45]. Our findings are however not in complete agreement with these results as the unaffected CK R and improved CK MA in our study suggest that the treatment effect is mediated by platelets and fibrinogen without enzymatic alterations in the coagulation cascade [45].

CK MA has previously been used as the primary TEG outcome parameter for CVD risk assessment [28–37]. Studies comparing CK MA in individuals with high vs. low CVD risk have shown similar differences as those observed in this present study. Gurbel et al. followed individuals after percutaneous coronary interventions and observed a 9 mm (12.5%) higher CK MA in individuals with recurrent events vs. no subsequent events [29]. Other studies have reported a 4.7 mm (7.2%) higher CK MA in individuals who underwent heart surgery vs. controls and a 5 mm (7%) higher CK MA in individuals with postoperative thrombosis vs. no postoperative thrombosis [33, 37]. In an infectious context, coronavirus disease 2019 (COVID-19)-positive pneumonias have been shown to induce a 4 mm (5.8%) higher CK MA compared COVID-19-negative pneumonias [35]. The severity of COVID-19 infections has also been shown to affect CK MA which was 7.8 mm (11.7%) higher in severe vs. mild COVID-19 [36]. In the present study, the corresponding changes were 6.1 mm (9.5%) and 2.7 mm (4.4%) in participants with TB and TBI, respectively. These changes are similar in magnitude to above mentioned results and it is therefore possible that treatment of TB and TBI might affect haemostasis to a degree which is enough to also reduce CVD risk.

The reduction of IFN-γ in response to TB treatment is in line with the findings of previous work [26]. Observations on the effect of TBI treatment on circulating inflammatory biomarkers are scarce and the present study is, to our knowledge, the first to describe reduced unstimulated levels of IFN-γ. In the only similar study, Zhang et al. measured cytokine markers in the unstimulated nil tubes used in IGRAs for TBI diagnosis without observing any change in IFN-γ after treatment [47]. The samples in the study by Zhang et al. were however incubated which makes comparison difficult.

Inflammation is a known driver of CVD and the reduced CK MA reported in this study could be explained by the accompanied decrease in inflammation [16–19, 21, 22]. Post-hoc correlation plots of the decrease in IFN-γ and CK MA showed a moderate correlation in the TB group (*r* = 0.502), but there was only a weak correlation in the TBI group (*r* = 0.217) which suggest that the relationship might be more complicated (data not shown). *Mtb* can reside in macrophages, which are known to be important drivers of atherosclerotic plaque formation, and there are many similarities between *Mtb*-induced granulomas and atherosclerotic plaques [48–50]. It is therefore possible that *Mtb* could contribute to CVD risk in a manner more similar to that of atherosclerosis via a more direct involvement of *Mtb*-infected macrophages.

### Strengths and limitations

The strengths of the present study include the prospective design and the fact that every participant was his or her own control which minimised individual variation and the impact of confounding factors. The exclusion of people with antithrombotic medication is also a strength since the antibiotics used for TB and TBI treatment, especially RIF, are known to interact with many antithrombotic medications [51]. Limitations of the present study include a low number of participants, the absence of a placebo control group and the lack of additional parameters associated with haemostasis such as fibrinogen levels. The exclusion of persons on antithrombotic medication may also be considered a limitation since we thereby excluded persons with high CVD risk. This is reflected by the low number of participants who were smokers and overweight. Thus, conclusions cannot be drawn regarding the impact of TB and TBI treatment on haemostasis in persons with high CVD risk. Another limitation is that four participants started new medications between baseline and follow-up. The majority of these are unlikely to affect our results, but the start of oral semaglutide in one individual with TB could be considered a confounder due to its beneficial effects on CVD risk [52]. The results were still significant for CK MA (*p =* 0.030) after post hoc analysis without this individual. RIF has in some studies been associated with thrombosis risk and this potential prothrombotic effect of RIF may have affected our results [53]. However, the majority of the participants were drug naïve at baseline and most follow-up samples were drawn on the last day, or the day after, treatment stop. The effect of RIF should therefore, if anything, have affected our results negatively. Another limitation is that we included four participants whose TB diagnosis was based on clinical and radiological findings suggestive of TB with clinical and radiological response to TB treatment. These participants could potentially have been infected with other Gram-positive bacteria such as Staphylococcus aureus which are normally sensitive to RIF. COVID-19 infections and vaccination during the study period may also have affected our results. Lastly, it should be noted that a positive IGRA reflects current but also former and cleared *Mtb-*infections. It is thus possible that the participants with TBI were treated without having an ongoing infection with viable bacteria.

## Conclusion

In this small study on adults treated for TB or TBI, TEG analysis showed that treatment was associated with reduced CK MA in both groups which suggest that there may exist cardiovascular benefits to treatment. Replication of our data, especially in persons with high CVD risk, could open up the possibility for systematic screening and treatment of persons with TBI as a way to prevent CVD.

## Electronic supplementary material

Below is the link to the electronic supplementary material.

Supplementary Material 1

Supplementary Material 2

Supplementary Material 3

## Data Availability

The datasets used and/or analysed during the current study are available from the corresponding author on reasonable request.
