# Correction: The effect of *Mycobacterium tuberculosis* treatment on thrombelastography-assessed haemostasis: a prospective cohort study

**DOI:** 10.1186/s12959-024-00670-z

**Published:** 2024-11-07

**Authors:** Hans Johan Niklas Lorentsson, Christina R. Clausen, Daniel Faurholt-Jepsen, Katrine Bagge Hansen, Sidse Graff Jensen, Rikke Krogh-Madsen, Per G. Hagelqvist, Pär I. Johansson, Tina Vilsbøll, Filip K. Knop, Pernille Ravn

**Affiliations:** 1 Section of Infectious Diseases, Department of Medicine, Herlev and Gentofte Hospital, University of Copenhagen, Hellerup, Denmark; 2 Center for Clinical Metabolic Research, Herlev and Gentofte Hospital, University of Copenhagen, Hellerup, Denmark; 3 Clinical Research, Steno Diabetes Center Copenhagen, Herlev, Denmark; 4 Department of Infectious Diseases, Rigshospitalet, University of Copenhagen, Copenhagen, Denmark; 5 Department of Clinical Medicine, Faculty of Health and Medical Sciences, University of Copenhagen, Copenhagen, Denmark; 6 Section of Respiratory Diseases, Department of Medicine, Herlev and Gentofte Hospital, University of Copenhagen, Hellerup, Denmark; 7 Department of Infectious Diseases, Hvidovre Hospital, University of Copenhagen, Hvidovre, Denmark; 8 Center for Physical Activity Research, Rigshospitalet, University of Copenhagen, Copenhagen, Denmark; 9 CAG Center for Endotheliomics, Rigshospitalet, University of Copenhagen, Copenhagen, Denmark; 10 Section of Infectious Diseases, Department of Medicine, Herlev and Gentofte Hospital, Hellerup Hospitalsvej 1, +45 38 67 38 67, Hellerup, 2900 Denmark

**Correction: Thrombosis J 21, 73 (2023)**

10.1186/s12959-024-00625-4

Following publication of this article, it has come to our attention that while reference 53 was cited in the text, it was omitted from the reference list due to a typesetting error.

The details of reference 53 are given below:

White NW (1989) Venous Thrombosis and Rifampicin. Lancet 334:434–435. 10.1016/S0140-6736(89)90603-X.

The original article has been updated.

