# Supplementary material for: The effect of Mycobacterium tuberculosis treatment on thrombelastography-assessed haemostasis: a prospective cohort study
Source: Thromb J. 2024 Jun 26;22:54. doi: 10.1186/s12959-024-00625-4 (PMC11201340; doi:10.1186/s12959-024-00625-4)
Supplement: Supplementary file 1 — Supplementary Material 1 [file 12959_2024_625_MOESM1_ESM.docx]

**Supplementary Table S1**: Lower and upper limits of detection for the V-PLEX Human Proinflammatory Panel I (4-Plex).

|  | **LLOD** | **LLOQ** | **ULOQ** | **Dynamic range** | **Unit** |
| --- | --- | --- | --- | --- | --- |
| **Analyte** |  |  |  |  |  |
| IFN-γ | 0.37 | 1.76 | 938 | 0.37-938 | pg/mL |
| IL-1β | 0.05 | 0.646 | 375 | 0.05-375 | pg/mL |
| IL-6 | 0.06 | 0.633 | 488 | 0.06-488 | pg/mL |
| TNF-α | 0.04 | 0.69 | 248 | 0.04-248 | pg/mL |
|  |  |  |  |  |  |
| IFN-γ, interferon-γ; IL-1β, interleukin-1beta; IL-6, interleukin-6; TNF-α, tumour necrosis factor-α; LLOD, lower limit of detection; LLOQ, lower limit of quantification; ULOQ, upper limit of quantification. | | | | | |
