# Supplementary material for: The effect of Mycobacterium tuberculosis treatment on thrombelastography-assessed haemostasis: a prospective cohort study
Source: Thromb J. 2024 Jun 26;22:54. doi: 10.1186/s12959-024-00625-4 (PMC11201340; doi:10.1186/s12959-024-00625-4)
Supplement: Supplementary file 2 — Supplementary Material 2 [file 12959_2024_625_MOESM2_ESM.docx]

**Supplementary Table S2**: Timing of biochemical measures in relation to treatment

| **Participant** | **Diagnosis** | **Days between treatment start and baseline samples** | **Days between treatment stop and follow-up samples** |
| --- | --- | --- | --- |
| 1 | TB | 18 | -92* |
| 2 | TB | 0 | 21 |
| 3 | TB | 0 | 12 |
| 4 | TB | 0 | 3 |
| 5 | TB | 0 | 26 |
| 6 | TB | 2 | -89* |
| 7 | TB | 0 | 2 |
| 8 | TB | 0 | -12 |
| 9 | TB | 0 | 8 |
| 10 | TB | -58** | 0 |
| 11 | TB | -33*** | 0 |
| 12 | TBI | 0 | 1 |
| 13 | TBI | -5 | 27 |
| 14 | TBI | 0 | 1 |
| 15 | TBI | 0 | 95 |
| 16 | TBI | 0 | 14 |
| 17 | TBI | 0 | 14 |
| 18 | TBI | 7 | 0 |
| 19 | TBI | 0 | 0 |
| 20 | TBI | 0 | 20 |
| 21 | TBI | 0 | 0 |
| 22 | TBI | 0 | 0 |
| 23 | TBI | 0 | 0 |
| 24 | TBI | 0 | 0 |
| 25 | TBI | 0 | 0 |
| 26 | TBI | 0 | 0 |
| Baseline and follow-up are set to time 0: Negative days mean that baseline/follow-up samples were taken before treatment start/stop. Positive values mean that samples were taken after treatment start/stop. *Participants who had samples taken after 6 months but had 9 months treatment planned. **The patient was initially diagnosed with TB based on clinical and radiological findings and had baseline samples taken at baseline. The patient did however decide to postpone treatment due to a longer vacation (microscopy and PCR were negative). During the vacation, sputum samples came back positive (few colonies) and started treatment on the return to Denmark. The initiation of TB treatment was therefore 58 days after the baseline samples. ***At inclusion, this patient was assessed as part of contact tracing, had a positive QFT and was planned to start treatment for TBI. However, clinical and radiological findings suggested TB or malignancy, and the subsequent diagnostic process delayed treatment initiation. The initiation of TB treatment was therefore 33 days after the baseline samples. TB, tuberculosis; TBI, tuberculosis infection | | | |
