# Supplementary material for: The effect of Mycobacterium tuberculosis treatment on thrombelastography-assessed haemostasis: a prospective cohort study
Source: Thromb J. 2024 Jun 26;22:54. doi: 10.1186/s12959-024-00625-4 (PMC11201340; doi:10.1186/s12959-024-00625-4)
Supplement: Supplementary file 3 — Supplementary Material 3 [file 12959_2024_625_MOESM3_ESM.docx]

Figure S1. Flow diagram of included participants

**TEG not taken after treatment**

Did not wish to participate (n =4)

Started oral anti-inflammatory therapy (n=1)

Misdiagnosis (not TB/LTBI) (n=2)

Lost to follow-up (n=1)

Delayed treatment start (n=1)

Discontinued treatment (n=1)

TB, tuberculosis disease; TBI, tuberculosis infection; TEG, Thrombelastography

**Included participants (n=51)**

TB: (n=24)

TBI: (n=27)

**Participants with TEG before treatment (n=36)**

TB: (n=16)

TBI: (n=20)

**TEG not taken before treatment**

TEG machine or cartridges unavailable (n=9)

Antithrombotic medication or oral birth control (n=5)

Known unavailability for follow-up visit (n=1)

**Complete TEG data before and after treatment included in this study (n=26)**

TB: (n=11)

TBI: (n=15)
